# Experience of the signaller explains the use of social versus personal information in the context of sentinel behaviour in meerkats

**DOI:** 10.1038/s41598-018-29678-y

**Published:** 2018-08-23

**Authors:** R. Rauber, M. B. Manser

**Affiliations:** 1 Animal Behaviour, Department of Evolutionary Biology and Environmental Studies, University of Zurich, Winterthurerstrasse 190, 8057 Zurich, Switzerland; 2 Kalahari Meerkat Project, Kuruman River Reserve, Van Zylsrus, Northern Cape South Africa; 3 Mammal Research Institute, University of Pretoria, Pretoria, South Africa

## Abstract

To maximise foraging opportunities while simultaneously avoiding predation, group-living animals can obtain personal information on food availability and predation risk and/or rely on social information provided by group members. Although mainly associated with low costs of information acquisition, social information has the potential to be irrelevant or inaccurate. In this study we use playbacks of individually distinct sentinel calming calls produced during sentinel behaviour, a form of coordinated vigilance behaviour, to show that meerkats (*Suricata suricatta*) discriminate between social information provided by different sentinels and adjust their personal vigilance behaviour according to the individual that is played back. We found that foraging group members acquired the lowest amounts of personal information when hearing social information provided by experienced individuals that act as sentinels most often in their group and littermates. Our study shows that social information can be flexibly used in the context of sentinel behaviour in order to optimize the trade-off between foraging and vigilance behaviours dependent on discrimination among signallers. We also provide novel evidence that the experience of sentinels rather than their age or dominance status is the main factor affecting the extent to which individuals use social information.

## Introduction

Accurate knowledge of an animal’s environment is crucial to ensure full exploitation of potential foraging opportunities while at the same time avoiding danger^1^. To acquire information on food availability and predation risk, individuals can assess the relevant environmental factors directly and gain personal information^2^. Additionally, group-living animals can use social information by monitoring other group members‘ behaviours and interactions with the environment^2–4^. Acquisition of social information allows faster and additional gathering of information, enhancement of skill learning, and lower costs of information acquisition for each group member^5–7^, resulting in fitness benefits for individuals using social information^3,8^. However, socially acquired information can be inaccurate, irrelevant or even deceptive leading to substantial costs for receivers^3,9,10^.

Discrimination of social information provided by different individuals might be particularly important in systems where callers have variable thresholds to call^11^ or where calls represent the relative risk the caller perceived during the calling bout^12,13^. As potentially inaccurate signals might come with the costs of an unnecessary predator response, or might have fatal consequences in case of not responding to a present predator^14^, it is expected that receivers of social information benefit from adjusting their responsiveness according to a signaller’s identity. In the context of alarm calling behaviour - a common source of social information - empirical research has shown that receivers of alarm signals in primates^15,16^ and squirrels^11,17–19^ are able to assess caller reliability by associating an individual’s identity with that individual’s past performances (e.g. the ability to discriminate between dangerous and non-dangerous threats and produce alarm calls accordingly). In these studies receivers differentiate between reliable and unreliable callers and adjust their response accordingly^11,15–19^, therefore lowering the potential costs of social information.

Sentinel behaviour is a form of coordinated vigilance behaviour, where mostly one individual scans the surroundings while the rest of the group is involved in other activities, mainly food acquisition^20–23^. By emitting sentinel calls, sentinels can provide the rest of the group with valuable, acoustic information about the presence of a sentinel guard^21,22,24^, the identity of the sentinel^21^ or the current, perceived predation risk^22,25^. Work on dwarf mongoose (*Helogale parvula*^26^) and pied babblers (*Turdoides bicolor*^27^) has shown that receivers discriminate between the quality (accuracy and relevance) of social information provided by different individuals and that the dominance status^26^, age^26^, group affiliation^28^ or perch height^27^ of the sentinels affect the extent to which other individuals rely on social information from them. However, previous studies have not been able to distinguish between the effects of variation in the signaller’s age, dominance status and experience in understanding the decisions individuals make when to use social versus personal information.

In meerkats (*Suricata suricatta*) an animal’s frequency of sentinel behaviour is not correlated with age, dominance status or group affiliation^29^, making them an ideal study species to determine the importance of these individual characteristics on the use of social information by other group members. Meerkats are small mongooses, which are naturally occurring in semi-desert areas of southern Africa. They are cooperative breeders, living in stable groups from three to 50 individuals, each group consisting of one dominant breeding pair and helpers^30^ (for details about dominance hierarchies and differences in the behaviour depending on social status within groups see^31,32^). This social system results in groups consisting mostly of full siblings (littermates) and half siblings. When foraging, meerkats mainly dig holes in the sand in search for insects and small vertebrates^33^, which prevents them from scanning their surroundings for predators. To minimize predation risk for the whole group, meerkats evolved an elaborate sentinel system with distinct sentinel calls^21^. Sentinel calls have been shown to contain information about the caller’s identity, as well as the sentinel’s perceived risk levels^21,25^. Sentinel calming calls, in particular, act as ‘all-clear’ signal eliciting an increase in foraging behaviour and a decrease in vigilance in other group members^25^.

In this study we investigated whether foraging meerkats differentiate between calming calls from different sentinels in their group and adjust their own vigilance behaviour accordingly. Specifically, we tested whether the dominance status, age, sex, sentinel frequency during the previous three months (as a proxy for experience), call rate (during sentinel behaviour) or whether the sentinel individual was a littermate (mostly full siblings) of the test subject predicted the extent to which receivers responded to the sentinel’s calls by decreasing their level of vigilance. As a consequence of the large variation in relative contribution to sentinel behaviour among individuals of the same group (mean = 8.3%, range = 1%–53%; unpublished data) based on sex^20,29^, dominance status^20^ and daily weight gain^29^ and since sentinel calming calls are individually distinctive^21^, we expect that foraging group members would discriminate between social information of different sentinels and therefore show according differences in their personal vigilance levels. In particular, we expected the vigilance levels of foraging group members to be reduced to a greater extent when they heard sentinel calls given by individuals that most often contributed to sentinel behaviour in a group (thereafter termed ‘super guards’) than when they heard calls given by individuals that less frequently (‘common guards’) or rarely (‘rare guards’) acted as sentinels.

## Results

**Figure 1:**
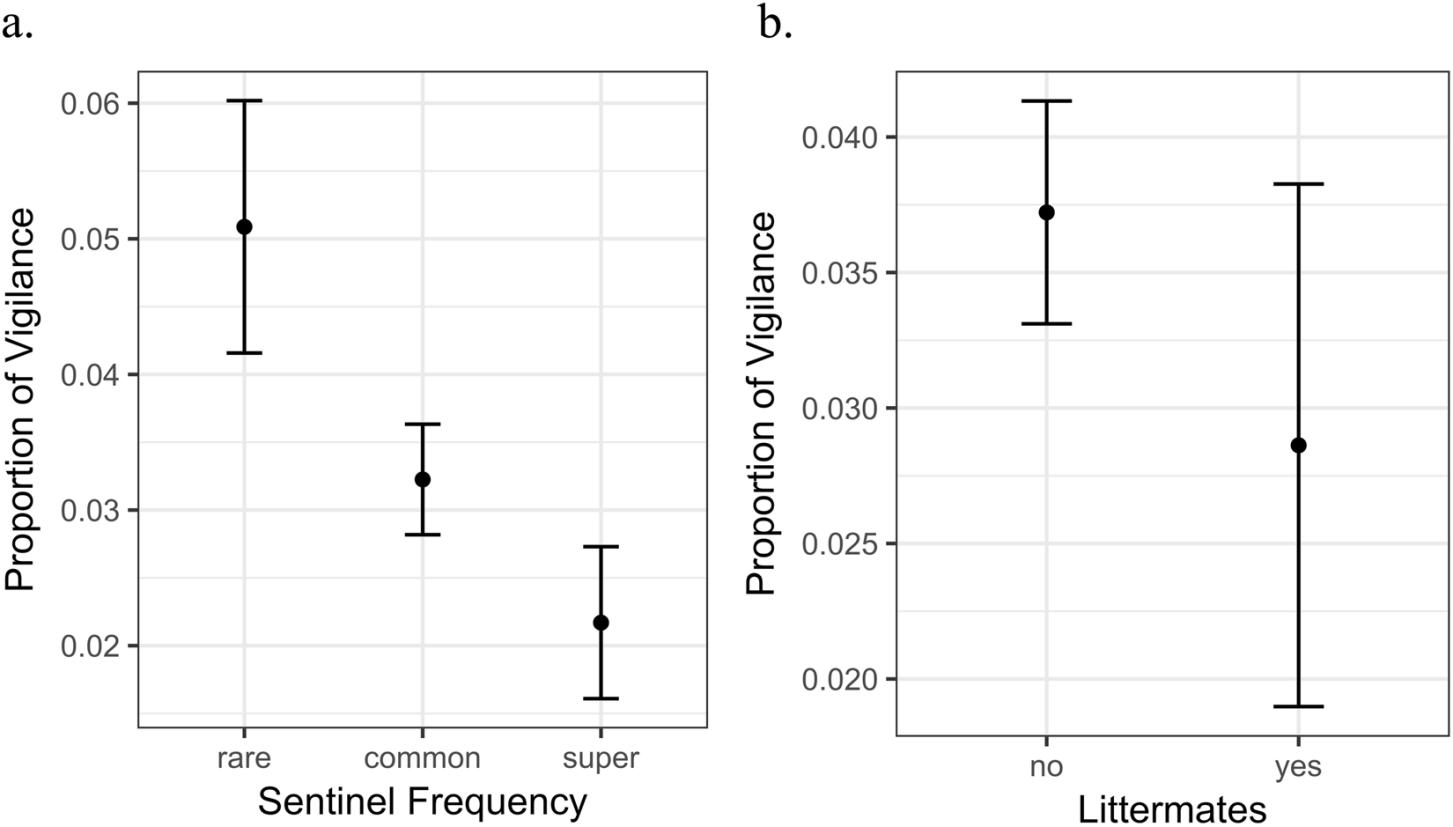
Influence of sentinel frequency and whether the sentinel is a littermate (mostly full siblings) of the test subject on vigilance levels. Proportion of vigilance in foraging test subjects in response to played back sentinel calming calls depending on (**a**) a sentinel’s frequency, and (**b**) whether the sentinel was a littermate of the test subject.

**Figure 2:**
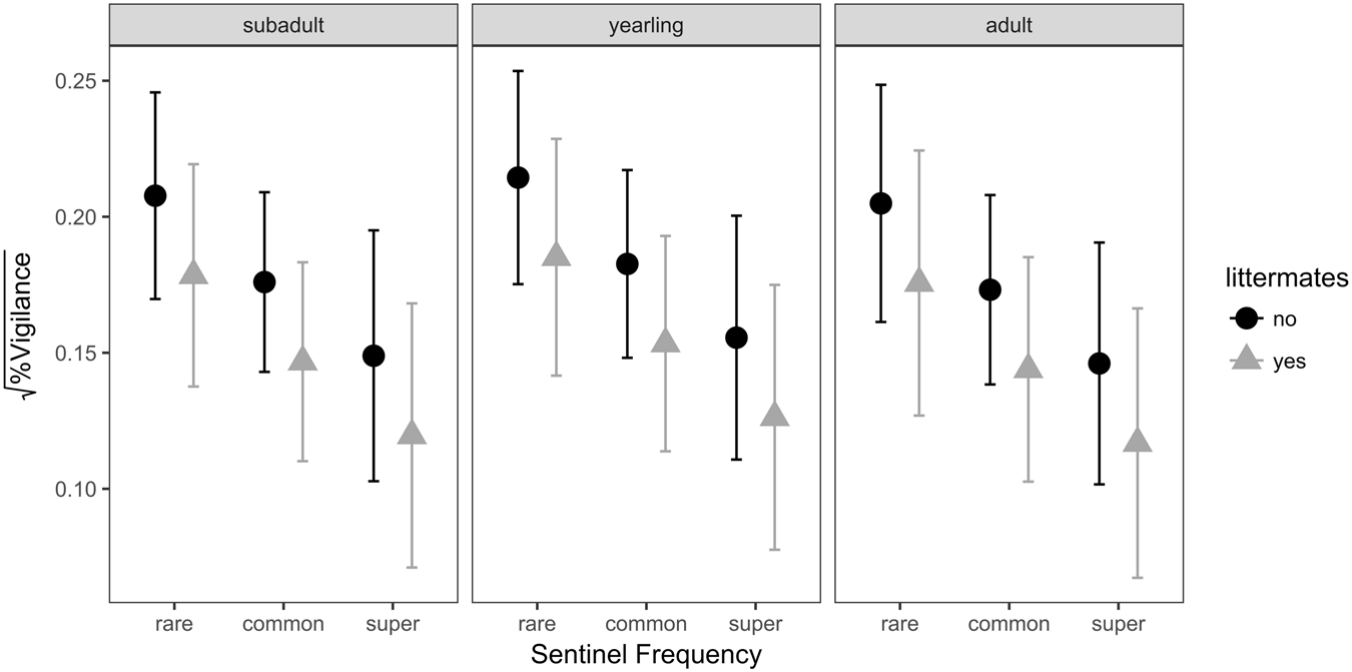
Influence of sentinel frequency, age category and whether the sentinel is a littermate (mostly full siblings) of the test subject on vigilance levels. Model estimates of the different vigilance levels (square root transformed data) based on sentinel frequency and whether the test subject was a littermate of the sentinel. The different boxes indicate the age categories of the sentinel that was played back.

Playing back sentinel calming calls given by different individuals to foraging test subjects in the same group revealed significant differences in the subjects’ vigilance levels depending on the identity of the sentinel (LRT: df = 65, χ^2^ = 10.12, p = 0.001). In particular, the frequency with which the caller contributed to sentinel behaviour during the previous three months and whether the test subject was a littermate of the sentinel significantly affected vigilance levels (Table 1, Fig. 1). Foraging test subjects spent the least time being vigilant, about 2.1%, when played the calming calls of the most frequent sentinels, the super guards, compared to calming calls of the common sentinels (3.2%; LMM: est = −0.025, se = 0.012, p = 0.070; Fig. 2) or rare sentinels (5.1%; post hoc multiple comparison: LMM: est = −0.051, se = 0.018, p < 0.001; Fig. 2). Calming calls from common sentinels elicited less vigilance behaviour than calming calls from rare sentinel guards (LMM: est = −0.026, se = 0.011, p = 0.013; Fig. 2).

**Table 1:** LRT showing which sentinel variables affected the proportion of vigilance shown by foraging test subjects.

Variable	DF	χ^2^ value	p-value
**Sentinel Frequency**	**2**	**12.18**	**0.002**
**Littermates**	**1**	**5.55**	**0.018**
Call Rate	1	3.78	0.052
Sex	1	2.64	0.125
Dominance Status	1	1.45	0.228
Age Category	2	0.48	0.786

We also found lower vigilance levels in test subjects in response to sentinel calming calls of littermates compared to playbacks of group members from different litters (LMM: est = −0.03, se = 0.01, p = 0.018; Table 1, Fig. 2). In addition, we observed a tendency for decreased levels of vigilance with an increase in call rate of the sentinel (LMM: est = −0.002, se = 0.0009, p = 0.052). Dominance status, age class and sex, however, had no effect on the proportion of vigilance behaviour shown by the test subjects (Table 1, Fig. 2).

## Discussion

Our study on meerkats shows that foraging group members discriminate between the calming calls of different sentinel individuals and adjust their personal vigilance behaviour accordingly. In particular, we found that foraging test subjects showed the lowest personal vigilance when hearing social information of individuals that acted as sentinel most often, and littermates. Dominance status, sex and age, however, did not have a significant influence on the observed vigilance levels.

The 50% reduction in vigilance levels between super and rare sentinels might indicate a shift from using social information towards personal information with decreasing experience of the caller. A more frequent sentinel individual might be better at assessing the current risk or the level of urgency coming from different types of threats. A likely explanation is that these individuals might be perceived to be more reliable, as they might have produced reliable signals in past predator encounters; however, this needs to be tested experimentally (for example with predator presentations coupled with playbacks). This would be in line with studies on yellow-bellied marmots (*Marmota flaviventris)* where the vocalisations of reliable alarm callers elicited the largest reduction in vigilance levels^19^ as well as studies on the dwarf mongoose that highlight the importance of reliability assessment of calls from different signallers in the context of sentinel behaviour^26,28,34^. However, whereas previous studies were unable to disentangle the effect of age, dominance status and experience on discrimination among signallers, our study shows that the frequency with which an individual has acted as sentinel in the recent past (experience), predicted the extent to which receivers use social information provided by that individual. This emphasises the importance to disentangle the roles of the relevant life-history traits, such as age and dominance status, from potentially correlated factors such as experience of the signaller in understanding the decisions individuals make when to use social versus personal information.

Our results imply that there must be some mechanism wherein meerkats can keep track of an individual’s contribution to cooperative vigilance behaviour, and thus, a sentinel’s experience levels. Association between sentinel calls of different signallers with these individuals’ sentinel frequency might act as simple mechanism to keep track of other group members’ contribution to sentinel behaviour. If littermates spend more time together, thus are closer in terms of social proximity, this could also explain why individuals showed less vigilance behaviour when they heard littermates compared to the rest of the group. Recent work on wild baboons *(Papio ursinus)* demonstrates the importance of social network parameters, such as the strength of social bonds, in explaining variation in the use of social information among individuals^35^. Alternatively, relatedness could affect the use of social information. Littermates are mostly full siblings while some of the other group members are half-siblings and very few individuals are non-related immigrants, raising the possibility that information provided by littermates (i.e. full siblings) might be valued differently than information from other group members. However, this needs to be tested further and future studies could apply a social network approach to test the influence of social and/or spatial proximity on personal vigilance levels. Lastly, foraging meerkats tended to be less vigilant when sentinel calming calls of individuals with higher call rates were played back. A likely explanation is that during longer silence intervals between calls individuals are more likely to gain their own information about the location of the sentinel guard as well as the current predation risk.

Together with previous work showing that meerkats do not discriminate between different alarm callers in their response to higher urgency aerial alarm calls^36^, our study highlights that discrimination among individuals based on vocal signals is highly call type and hence call function dependent. Both call types, alarm calls and sentinel calls, are typically produced by the individual on sentinel guard, with sentinel calls relating to the perceived predation risk^25^ and alarm calls, in case of a detected predator, to predator type and urgency level^37,38^. While it has been argued that alarm calls are too costly to ignore^36^, sentinel calming calls provide more flexibility in the response of the receivers. Here the costs of a wrong assessment are not as severe as when ignoring an alarm call, but still high enough for natural selection to favour receivers which maximise their foraging intake by discriminating between different caller’s quality of social information and adjust their vigilance behaviour accordingly. Our study suggests that the urgency of a correct response predicts whether receivers trade-off the accuracy and relevance of their own versus social information and leads to behavioural adjustment of the receivers.

Across a wide range of species of fish^39^, birds^40^, and mammals^11,26,28^, group living animals constantly assess the accuracy and relevance of social information and consequently shift their own behaviour between relying on social information and obtaining personal information. Other studies established that the signaller’s individuality^11^, age class (for example^18,19,41,42^), dominance status^26^ or group affiliation^28^ are key factors assessing the reliability of social information by the receivers. Our study demonstrates the adjustment of personal vigilance behaviour by receivers dependent on the experience of the signaller, which may also represent the assessment of reliability of sentinels. Hence, depending on the context, discrimination among individuals might be based on different attributes of the signaller, allowing other group members to flexibly maximise acquisition of the highest quality information, be that social or personal information. Based on our results that age or dominance status are not necessarily an accurate proxy for the information value of an individual’s signal, we emphasize the need in future studies to disentangle these factors from experience whenever possible. Our study demonstrates how social information can be flexibly used to optimize the trade-off between foraging and vigilance behaviours dependent on discrimination among signallers.

## Methods

### Study site and population

This study was carried out between March and June 2016 and March and May 2017 at the Kalahari Meerkat Project, Kuruman River Reserve in the southern Kalahari Desert, South Africa. The study site has a semi-arid climate and is characterised by perennial grasses, shrubs and trees as main vegetation (for more information about habitat and climate at the study site see^30,33,43^). All meerkat groups used in this study were habituated to close human observations and to the sound recording and playback equipment, allowing to conduct the recordings within a distance of 0.2–1 m from the calling meerkat. All necessary information about individual identity, age, dominance status and sentinel frequency (number of events when the individual acted as sentinel) was collected as part of the long-term data collection of the Kalahari Meerkat Project. We used nine groups that ranged in total group size from three to 23 individuals per group for the playback experiments (mean group size ± sd = 10.28 ± 4.77). According to their frequency of exhibiting sentinel behaviour, our proxy for sentinel experience, we assigned all individuals of the same group relative to each other into three categories: rare (below average group contribution), common (above average group contribution) and super guards (>50% than the second most frequent sentinel in a group). As the frequency with which an individual acts as sentinel is likely to change over longer periods of time, we based this categorisation on an individual’s sentinel frequency over a range of three months prior to the playbacks. This time span of three month was chosen because nutritional and reproductive state affects an individual’s probability to go on sentinel guard^20,29^ and thus should be rather consistent during this time interval. Duration of the sentinel bouts did not differ between the three different sentinel categories (ANOVA: df = 2, F = 1.12, p = 0.34).

### Acoustic recordings

We recorded sentinel vocalisations from 66 different individuals (3 to 14 individuals / group) produced during natural sentinel bouts no more than three months prior to the playbacks using a Sennheiser directional microphone (ME66/K6) connected to a Marantz PMD-670 solid-state recorder (Marantz Japan Inc.). Whenever the sentinel was calling from a tree or any other position difficult to access, the microphone was fixed on a telescope pole in order to keep the recording distance at less than 0.5 meters and thereby maintaining a high signal-to-background ratio. From these recordings we created spectrograms in Adobe Audition CC (2015.0 Release) to assign each call to one of the six sentinel call categories described by Manser (1999). Previous work on meerkat sentinel calls showed that visual inspection of the spectrograms enables for accurate assignment of the different sentinel call types^44^. Playback files consisted of sentinel calming calls and background noise that were cut out from natural recordings and pasted into a new sound file using Cool Edit Pro software (Syntrillium Software Corporation). Only sentinel calming calls, i.e. “single note” and “double note” sentinel calls, were used for the playbacks, as these calls were shown to have an “all-clear” function and typically lead to a decrease in vigilance and an increase in foraging behaviour in receivers^25^. The call rate of these calming calls within the playback was kept the same for all playbacks of the same individual as during the natural recordings (mean call rate ± sd of all individuals: 13.41 ± 6.22 calls/min, range: 4–34 calls/minute). Natural variation in call rate among individuals allowed us to test if call rate itself affects vigilance levels rather than the individual identity. The use of individual call rates for each played back sentinel (calculated by averaging the call rates of three to five recordings of natural sentinel events) simulates the presence of a specific sentinel as naturally as possible and therefore avoiding any vigilance response by the receivers due to unnatural conditions. There was no significant difference in the call rates of individuals from the three different sentinel frequency categories (ANOVA: df = 2, F = 1.48, p = 0.23) or littermates versus non-littermates (ANOVA(df = 1, F = 0.057, p = 0.81). Each playback file consisted of calls from at least three different recording events. The duration of playback files was five minutes in total.

### Playback Experiments

To play back the sentinel calming calls, we used an iHome rechargeable mini speaker (iHM79SC), which was kept at 1 m height, representing the average location height of an individual on sentinel. Amplitude of each playback was “by ear” adjusted to natural weather and wind conditions of naturally occurring sentinel calls and thus changed according to environmental conditions. Over both seasons a total of 544 playbacks were conducted to single foraging test subjects at a time. In groups where we were able to record all individuals in order to compile sentinel playbacks, every individual’s sentinel calming calls were played back to each group member (test subject). Whenever this was not possible because we did not get any or not enough sentinel recordings from some individuals in the group (hereafter called “non-guarders”), we ensured that these “non-guarders” were also used as test subjects for the playbacks to avoid any biases in the responsiveness. Moreover, when the group was too big (>10 individuals older than 6 months) to test every group member, we always chose at least one “guard” and one “non-guard” per age class, including one male and one female for mixed sexed litters to account for differences in responsiveness between different age classes and sexes. This resulted in a total sample size of 544 playbacks from n = 66 sentinel individuals conducted on 112 foraging test subjects (n = 66 “guards” and 46 “non-guarders”). To avoid habituation to a specific track of an individual to several test subjects in the same group, we repeated it three times at most over a time period of 3–3.5 hours. To keep external effects minimal we always played 3–4 different playback tracks to the same individual and then switched to another test subject. Therefore, the time interval between playing the same playback track was at least 30 minutes.

Playbacks were only conducted during morning foraging session and were only started when no predator was in sight, the majority of the group was continuously foraging for at least 10 minutes and at the same time no natural sentinel was up (to avoid any interferences with other sentinel calls). In case of an approaching predator or one group member going on sentinel guard, the playbacks were paused and only resumed when the previously mentioned conditions were met for at least five minutes. To get a natural amount of exposure to sentinel calls the natural frequency of sentinel behaviour has been calculated based on data from the same nine groups during March to June during three previous years. The average time a group has a sentinel on guard is around 71 minutes during a 180 minutes observation session (mean ± sd = 71.25 ± 49.74). Therefore, it was possible to conduct up to fourteen five-minute playbacks (mean number of experiments ± sd = 8.6 ± 3.6) in one morning foraging session lasting typically 3–3.5 hours, ensuring a natural exposure to sentinel calming calls (groups were visited once a week at most to avoid habituation to experiments).

### Behavioural observations

Simultaneously to the five minutes of the playback experiments behavioural observations of the test subjects were continuously recorded using the behaviour-tracking program Cybertracker (Cybertracker Conservation Version 3.479) installed on an Acer tablet (IconiaOne 7 B1–750). From these behavioural observations we calculated the proportion of vigilance behaviour (quadruped and bipedal scanning of the surroundings) by the subject during the five minutes of the playbacks (total seconds of vigilance behaviour / 300 seconds).

### Statistical analysis

All statistical analyses were done using R v2.1 (R foundation for statistical computing). We fitted a linear mixed effects model (LMM) to test the relationship between the proportion of vigilance behaviour during the playbacks and the variables of interest. Sentinel individual nested within group, test subject nested within group, as well as date nested in year were used as random effects. To see which characteristics of a sentinel individual affect vigilance levels of the receivers, we used dominance status, age, sex, previous sentinel frequency (as a proxy for experience), call rate and whether the test subject was a littermate of the sentinel as fixed effects. All LMM were run using the lme4 package^45^. To determine whether the fixed effects had any significant effect on the response variable, we used likelihood ratio tests (LRT) to compare whether the model with the fixed effect included differed significantly from the same model with the fixed effect excluded^46^. We tested for any interactions between sentinel frequency and sex, age category and dominance status but removed them again because of non-significance. Normality of the data was determined by examining diagnostic plots. The response variable was square root transformed to normalise the residuals in order to meet the assumption of the LMM. LmerTest package was used for contrasts within the model, whereas multiple comparison tests with manually set contrasts were used to compare the different categories not specified by the intercept^47^.

### Ethics

All research for this study was conducted with permission of the ethical committee of Pretoria University and the Northern Cape Conservation Service, South Africa (Permit number: EC031-13). All the methods were carried out following the approved guidelines.

### Data availability

The data are available from the corresponding author on reasonable request.
